# Dr. Kinglake on Arsenic in Herpetic Affections

**Published:** 1813-07

**Authors:** Robert Kinglake

**Affiliations:** Taunton


*To the Editors of the Medical and Physical Journal.*

GENTLEMEN,

ARSENIC has been long known to be a powerful agent in medical practice, but its employ has been accompanied with too much dread of its proving deleterious, to have allowed of its confident and adequate use. Its reputation for being efficacious in leprous, syphilitic, and other affections, as recorded by Swediaur,* and others, has

---

* Swediaur, on the internal Use of Arsenic, says, "Apud Hindous forma pilularum: Elephantiasis vera seu lepra nigra, aliæque cachexiæ; neura desparata; morbi syphilitici rebelles; morbi ab hydrargyro producti; paralysis; febres intermittentes rebelles."

stood stood on an authority too vague and equivocal to have gained it sufficient trial. It is to inveterate cases of disease that is owed the discovery of some of the most valuable remedies, as in those instances agents of extraordinary powers are resorted to as a last attempt to subdue untractable distempers. In these circumstances, the substance under consideration has been occasionally subjected to the test of experiment; and, if the result has been favorable to its salutary efficacy, it either has or ought to have been recorded as useful practical information. In this way arsenic has been lately employed, under my direction, in three most obstinate cases of herpetic affection.

In the first instance, the disease assumed a distinct eruptive form, having broad circumscribed inflamed bases, similar to the character of herpes, denominated *pemphigus.* Much pain attended the progress of these eruptions, some of which would imperfectly suppurate, burst, and discharge an ichorous fluid; others would remain comparatively indolent, putting on a livid hue, and gradually lose their inflamed and tensive hardness without maturating. After the lapse of a few weeks, a fresh eruption would succeed the former, and in this course the complaint had continued for several months at the time of my being consulted.

It never had occurred to me to see an instance of this affection but once before, which was in the Imperial Hospital at Vienna, where it was shown to medical spectators as an object of curiosity rather than of cure. It had indeed resisted every mode of treatment that had been instituted for its removal, and was at that time considered as an incurable disease. The recollection of this fact caused me to despair of deriving any aid from common agents, and induced me resolutely to try what an adequate use of arsenic would effect. The patient was accordingly directed to take ten drops of Fowler's solution of that substance three times a day. This was continued during about three months without any sensible inconvenience to the general health, and without much apparent amendment of the complaint. So little benefit, indeed, was thought to have been obtained from it, that it was at length discontinued. In the course of a few weeks after, however, the eruptions ceased to be renewed, and those that were on the surface became progressively less troublesome, until they totally subsided, leaving only the skin discolored under which they were situated. The patient was, of course, much gratified by the unexpected disappearance of the afflicting disease; but the interval from leaving off the medicine to the cessation of the complaint, having been some weeks, the relief was erroneously supposed supposed to have arisen rather from the discontinuance than from the efficacy of the remedy. That this was not the case, the long duration and uniform continuance of the complaint for nearly twelve months, although assiduously treated by various medicines of reputed virtue, made it sufficiently evident; yet the truth of this opinion was rendered additionally clear by the analogous efficacy of the medicine in the subsequent case of herpetic affection.

In this instance, the affection was characterised by extensive blotches, scarcely elevated above the surface of the skin. These appearances had proceeded through the eruptive and drying stages, with a sense of burning heat in the former, and of intolerable itching in the latter, during a series of several years, in which time, no mode of remedy that either theory or practice could suggest had been untried, but all proved equally unavailing. The general health was not sensibly disordered, but the habit of the cutaneous disease seemed to be obstinately established. On my being consulted on a complaint that had existed so long, and had baffled the various means of relief that had been devised for its removal, it appeared to me that it was a suitable occasion for a full trial of the curative powers of arsenic: it was therefore directed, as in the former instance, in doses of ten drops three times a-day, and continued for about six weeks without any inconvenience to the general health, and without any manifest advantage to the cutaneous disease. It was now discontinued as a medicine not more efficacious than others that had been previously tried, and in utter despair of finding any remedy for so inveterate an ailment. Thus disappointed and discouraged, the disease was abandoned to its customary course, when, after a few weeks, it was observed that the blotches were less visible, and had lost much of their usual redness, heat, and itching. This welcome change afforded much gratification, which continued to be heightened by a progressive amendment, until the face became free from all fresh eruptions, and nothing more than a slightly discolored hue of the skin formerly affected remained of the disease. This amendment was not temporary; it continued, and soon evinced, by the natural aspect of the portions of skin that had been so long and so severely diseased, that the healthy state had been restored, and was likely to be permanently established.

The close analogy subsisting between these two cases, respecting the salutary influence of the agent not being shown until after its use had been discontinued for some time, evinces that a similar curative process had obtained in each. It is probable that the high stimulating and tonic effect effect of the remedy whilst using, is such, as to prevent its beneficial operation from being observable; that, although the diseased action against which the medicine had been directed might be overcome, yet the arsenical excitement of the part would remain equally violent, and be mistook for an unaltered continuance of the original affection. If this be the fact, it suggests a very useful practical rule in the management of this powerful medicine, that of giving it for a limited period, and then suspending its use, if active irritation should still continue, with a view to ascertain to what source such morbid action be referable.

That the curative influence of arsenic may be thus disguised, is rendered additionally probable from a similar effect occasionally arising in the use of mercury. It has often occurred to me to see the most ill-conditioned sores in syphilitic cases, that had been subjected to excessive mercurial treatment, in which, very naturally, the farther the medicine was urged, the more phagedenic and eroding the ulcers became. In these circumstances, the suspension of the medicine alone is in general found to effect the desired change, by removing the only obstacle to healthy healing. The analogy does not stop here; it promises to be found an important pathological fact, resting on a physiological principle in animal life, that, although one action might be rendered so powerful as to overcome another, yet that the ascendant one will equally precede the resumption of the healthy state, if sustained by an unremitted application of its influence. When approved remedies have been long employed without producing the usual or desired effect, it would be advisable (especially in chronic affection of no great emergency) to suspend their use for some time, until any conceivable inordinate influence excited by them may cease, and an opportunity of recovering the healthy state be thus afforded to the native resources of the diseased parts.

Many of the severest diseases to which human nature is incident, find spontaneous cures in the natural exhaustion of that degree of vital energy which sustains their temporary existence. Of this class are the different kinds of febrile affections, inflammatory disorders, and indeed a considerable portion of those even that are referred to a vitiated state of healthy action, and are consequently deemed indispensable objects of medicinal treatment. But the duration of natural cures is too indefinite to be quite safe: the issue between the period in which they may be effected, and the destructive injury which might in that time be inflicted on the fundamental conditions even of vital action, is often too perilous to be risked. The best powers of art are therefore, on those occasions, justly demanded to accelerate the terminationof a state of disease that can admit of no long delay compatibly with life. Such states of disease often require the most powerful exertion of medicinal influence, an extent of efficiency indeed only to be measured by the limit of safety in the employ. Of this description is arsenic in the herpetic diseases recited. It was used under circumstances in which other powerful medicinal agents had no curative effect. It had been found to be a safe medicine, and the result proved that it ultimately subdued a state of morbid action which appeared to be beyond the reach of other modes of relief.

In a third instance, arsenic has also very lately supported its claim to curative efficacy in herpetic affections. This was a case of inflammatory eruptions confined to the hands, which would tumefy and proceed to maturation, accompanied with extreme pain; and after this diseased course had terminated, it would be renewed, running a similar career; and so it had gone on in many succeeding instances during several months. No mode of treatment was found to avail, until, by my advice, arsenic was freely taken. From ten to twenty drops of the solution before-mentioned were administered for upwards of three months, when the sores were imperfectly closed, assuming a scurfy appearance. An ointment composed of half a dram of arsenic finely levigated, and one ounce of hogs-lard, was ordered to be applied to the pustular surfaces; this sometimes occasioned acute pain, and at others, according to the substance of the intervening scale of scurf, it was borne without inconvenience. Visible amendment was produced in the course of a fortnight, but it was not until after four months that the prevailing disposition to a renewal of irritation was finally overcome; nor, as in the former cases, did the cutaneous redness and scurf disappear until some months after the discontinuance of the medicine. These lingering vestiges of disease at length, however, wholly subsided, and the patient has since (now a considerable period of time) remained perfectly free from the complaint. No injury arose to the general health during the long use of this medicine, which affords a conclusive proof of the safety with which a full and unsparing trial of its powers may be pursued.

In a fourth instance, arsenic has been employed under my observation, both internally and by friction,* to a vast extent,

* The mode of attempting to introduce arsenic into the system by friction was at that time novel, at least it was not then known to have been tried. The patient was disposed to adopt the expedient from tent, during several months, with a view to overcome the supposed remains of syphilitic disease. The complaints of pain and swelling in different bones were sensibly mitigated by its influence, but not wholly subdued. Visceral disease appeared to be an obstacle to its more extended efficacy. It soon induced so much distress throughout the region of the liver as to render its farther use intolerable. This became an insuperable bar to a full trial of its powers. The patient, who is a medical practitioner, is firmly persuaded that it would accomplish his curative object if the state of the hepatic organ would permit its efficacy to be carried to the requisite extent.

Enough seems to be known of this powerful medicine to entitle it to high consideration in medical practice. Its *modus operandi* appears to be that of a most pervading stimulant, in which mode of acting it also produces a sensible tonic effect. Patients debilitated by the vexatious and harassing continuance of herpetic affections, have the tone of the system at large improved by arsenical influence. This may be regarded as a most valuable circumstance in its medicinal efficacy, as it is adapted to secure and render permanent any altered action that its salutary agency may produce. Its tonic power in obviating the recurrence of intermittent fever has been evinced, and it has been also affirmed to be exceedingly beneficial in sustaining and renovating the general strength in low remittent fever; but my own experience of it in that disease has induced me to consider it

---

from the circumstance of the stomach having been so nauseated by its internal use, that it could not be longer borne in that way with any degree of convenience. A tea-spoonful of the arsenical solution was triturated with an ounce of the saponaceous liniment, half of which was rubbed in on different parts of the body, more particularly on the arms and legs, twice a-day. The effect produced on the morbid sensation that prevailed, by those frictions, was precisely what resulted from the internal use of the medicine; but, after the friction had been continued about a fortnight, the salivary glands became affected much in the same manner as is usually occasioned by a protracted use of mercury. The friction was then suspended, and the inordinate excitement of the salivary glands ceased. The external employ of arsenic was thus pursued during at least six weeks, and appeared uniformly to produce similar, and indeed in the opinion of the patient more powerful effects than were found to arise from its internal exhibition. To know that this active remedy might be made effectually to reach the system by external application, is of practical value when its virtues may be indicated, and the state of the stomach be such as not to bear the direct impression which its peculiar medicinal influence is adapted to produce.

 as as hurtfully stimulant, yet it must be confessed that my occasions for trying it in that intention have been much too limited and equivocal to warrant a decided opinion on the subject.

To have it clearly ascertained that a most powerful agent has operated an effectual cure in a class of diseases usually very unyielding, and that it may be given freely with perfect safety, is practical knowledge of extensive application, and may, under skilful direction, be turned to incalculable benefit.

The preceding observations on the salutary efficacy of arsenic in herpetic diseases, were written upwards of two years since, and would have been then published but for continued opportunities that were presenting of extending the proof of its virtues to a degree that would leave no doubt of its remedial powers in the cases in which it had been employed. Since that period, an accession of experience has been obtained that warrants the utmost confidence in its curative powers.

It would be tedious to state in detail, as in the foregoing narrative, the particular cases in which it has succeeded; it is sufficient to affirm, that it has rarely failed to produce the desired effect, and that too in a way that has been unattended by any unpleasant occurrences, and with manifest advantage to the general health. Its medicinal action has been uniformly that of a powerful tonic, so much so, as in some instances to induce visceral excitement either on the brain, lungs, or liver, but not in a degree that did not speedily subside on discontinuing its use for a few days.

As before observed, its curative influence was not apparently shown, in many cases, until some weeks after its use had been relinquished; and in several instances in which the occurrence of visceral excitement became an impediment to its farther employ, the herpetic disease, for which it had been given, permanently subsided during the interval of its disuse, so as to render its farther administration unnecessary. It may be fairly a question, whether taking of arsenic for an indefinite length of time may not be productive of injury. The arsenical action that is induced may, by its tonic influence to a given extent, prove highly beneficial in the circumstances of systematic debility commonly existing in herpetic diseases; but when that influence is carried beyond the healthy limit of action, it may go to inflammatory excitement, and to the various mischief resulting from that morbid state: it therefore would at once be most safe, and apparently most conducive to the early and decided efficacy of the medicine, to give it during about a month, and then to intermit intermit its use for an equal length of time, when it might be resumed if the disease should not in the interim have wholly disappeared, which has actually been the case in a large majority of instances under my observation.

It has not occurred to me to know that either any immediate or distant injury has resulted from the use of arsenic; and if it be given in doses from one to twenty drops three times a-day for about a month, and then discontinued, and resumed, if necessary, as before mentioned, no deleterious consequence will ever be likely to ensue from it. It has been freely taken under my direction from the early age of three months throughout the various intermediate periods to advanced life, and in no instance has it occasioned any inconvenience that would warrant imputing to it the character of being a dangerous remedy. That it is a very powerful medicine there can be no doubt, but its power is exerted in a way that would seem to be perfectly safe in every variety of temperament, and under all the different circumstances of herpetic affection in which it has been hitherto employed. A medicine sufficiently active to subdue herpetic disease, must be capable of extensive influence over various morbid conditions of life; and it is probable that the highly tonic and pervading operation of arsenic, will hereafter be found of vast importance in the cure of many of the most inveterate diseases incident to human nature. I am, &c.

*Taunton, May* 4, 1813. ROBERT KINGLAKE.

